# Novel Insights into Insect-Microbe Interactions—Role of Epigenomics and Small RNAs

**DOI:** 10.3389/fpls.2016.01164

**Published:** 2016-08-04

**Authors:** Dohyup Kim, Margaret W. Thairu, Allison K. Hansen

**Affiliations:** Department of Entomology, University of Illinois, Urbana-ChampaignUrbana, IL, USA

**Keywords:** symbiosis, epigenomics, DNA methylation, small RNAs, insect-plant interactions

## Abstract

It has become increasingly clear that microbes form close associations with the vast majority of animal species, especially insects. In fact, an array of diverse microbes is known to form shared metabolic pathways with their insect hosts. A growing area of research in insect-microbe interactions, notably for hemipteran insects and their mutualistic symbionts, is to elucidate the regulation of this inter-domain metabolism. This review examines two new emerging mechanisms of gene regulation and their importance in host-microbe interactions. Specifically, we highlight how the incipient areas of research on regulatory “dark matter” such as epigenomics and small RNAs, can play a pivotal role in the evolution of both insect and microbe gene regulation. We then propose specific models of how these dynamic forms of gene regulation can influence insect-symbiont-plant interactions. Future studies in this area of research will give us a systematic understanding of how these symbiotic microbes and animals reciprocally respond to and regulate their shared metabolic processes.

## Introduction

Microbial associates that interact with insects can produce a wide array of metabolic products that complement the metabolic needs of their herbivorous hosts (Hansen and Moran, 2014). Consequently, microbes that form persistent but noninvasive associations with their hosts have the potential to provide their hosts with useful novel gene products in a short evolutionary timespan. How these symbiotic microbes and animals reciprocally respond to and regulate shared metabolic processes is a nascent but emerging area of research.

Animals, including insects, can biosynthesize some but not all of the amino acids that are required for building proteins. Food sources that are deficient in those essential amino acids (EAA), such as plant sap, present a nutritional challenge to consumers. Most insect herbivores that feed on a phloem or xylem sap diet are able to feed on this essential amino acid-deficient niche because they harbor one or more nutritional symbionts (Hansen and Moran, 2014). One model system that has been productive for teasing apart the regulatory mechanisms of shared animal-microbe metabolic processes is the pea aphid, *Acyrthosiphon pisum*, a sap-feeding insect in the order Hemiptera, and the bacterium *Buchnera aphidicola*, the mutualistic endosymbiont found in most aphids. Aphid and *Buchnera* physiologies are integrated for the production of amino acids and this occurs within specialized aphid cells called bacteriocytes. Specifically, *Buchnera* relies on the aphid for the biosynthesis of aphid-encoded non-essential amino acid pathways, and the aphid relies on *Buchnera* for the biosynthesis of *Buchnera*-encoded essential amino acid pathways. Previous work on this system supports the prevailing hypothesis that this integrated metabolism is regulated primarily by the aphid host via aphid-encoded transporters (Price et al., 2014) and aphid genes that complement *Buchnera*'s EAA pathways (Hansen and Moran, 2011; Poliakov et al., 2011). Moreover, an aphid-encoded protein of bacterial origin can be transported into *Buchnera* cells and therefore a cross-domain protein translocation system exists for this intimate symbiosis (Nakabachi et al., 2005, 2014).

Gene expression of aphid bacteriocytes has been characterized at the transcriptome and proteome level (Hansen and Moran, 2011; Poliakov et al., 2011). As expected pathways involved in the amino acid metabolism are especially enriched in bacteriocytes compared to other aphid body cells (Hansen and Moran, 2011; Poliakov et al., 2011). However, the regulatory factors that lead to the development of these tissues and their signature expression profiles are not well-understood. In other animal systems, the primary regulatory factors that determine a eukaryotic cell's fate and its potential reprograming include histones, DNA methylation, noncoding RNAs, and transcription factors (Peter and Davidson, 2015). Work by Braendle et al. (2003) did identify three transcription factors that are expressed in temporal order, Dll, En, and Ubx or Abd-A, during bacteriocyte development in aphid embryos. The timing and expression of this subset of transcription factors is unique compared to any other cell type in insect embryos (Braendle et al., 2003). Currently it is unclear how these transcription factors may regulate metabolic processes or if other unknown co-factors are involved during embryonic stages or later during maternal bacteriocyte development. Moreover, it is unknown if chemical marks on histones and/or DNA (i.e., epigenetic mechanisms) (Hansen and Moran, 2014) are important in the regulation and metabolic reprogramming of these symbiotic cells, especially in response to environmental signals such as host plant nutrients or secondary plant compounds. Therefore, further understanding of how different subsets of host genes turn on and off in bacteriocyte development in response to environmental stimuli is required in order to fully understand how these intimate symbioses evolved, how they are maintained, and how they may ultimately influence host-plant-interactions.

Although evidence from the aphid and *Buchnera* model system suggests that the host exerts the majority of the regulatory control on this symbiosis, the role of the symbiont in gene regulation is not well-defined (Hansen and Moran, 2014). Given that *Buchnera* has lost the majority of the canonical mechanisms for gene regulation its capacity to be a participant in gene regulation has long seemed compromised (Shigenobu et al., 2000). Nevertheless, it was recently suggested that *Buchnera* regulates its own protein expression via putative post-transcriptional mechanisms (Hansen and Degnan, 2014). This recent study presents somewhat of a paradigm shift in understanding the regulation of these intracellular host-microbe mutualisms. It is now clear that symbionts with reduced genomes can potentially regulate their genomes through post-transcriptional processes, such as through regulatory small RNAs (sRNAs; Hansen and Degnan, 2014). Consequently, the potential role of microbe-mediated gene regulation cannot be ignored in these shared insect-microbe regulatory networks.

Recent advancements in the field of molecular biology and genome sequencing technologies allow for the first time the ability to predict how epigenetic mechanisms and regulatory sRNAs may impact the regulation and evolution of animal and microbial genomes. This review draws upon these emerging fields of gene regulation to investigate the potential role of epigenetics and sRNAs in *both* insect and microbe-mediated regulation of shared metabolisms. By understanding the dynamics and the intersection of these regulatory mechanisms, we can begin to disentangle the evolution of these shared herbivore-microbe metabolisms. Ultimately, if we can identify key molecular mechanisms that are responsible for regulating shared amino-acid metabolisms, which are widespread in symbiont-enabled herbivory, we can determine if the evolution of these mechanisms impacts insect-plant interactions.

For the first part of this review we will discuss previous literature on epigenetic mechanisms in eukaryotic gene regulation, and how epigenetics are potentially involved in herbivore-microbe interactions. For the second part of this review we will discuss previous literature on regulatory RNAs and how they are potentially involved in the regulation of these insect-microbe interactions.

## Gene regulation via epigenomic mechanisms

Under Darwinian natural selection random genetic mutations in the DNA molecule are inherited from parent to offspring, and increase in frequency within a population if a given mutation contributes to differential reproductive success (Dobzhansky, 1937). Alternative mechanisms of adaptation, such as Lamarkian inheritance, where an individual can pass down acquired traits that are obtained during its lifetime, have been hotly debated (Pilpel and Rechavi, 2015). Evolutionary theory that involves different variations of Lamarkian inheritance has been resurrected multiple times through history (Burkhardt, 2013). One controversial variation of Lamarkian inheritance, neo-Lamarkism (Skinner, 2015), has been proposed to explain epigenetic inheritance (Jablonka and Lamb, 2015) and CRISPR-*cas* immunity in Bacteria and Archaea (Koonin and Wolf, 2009), because these acquired traits are not random but are induced by the environment in a predictable fashion and are inherited through generations.

Epigenetic marks referred to here as chemical marks on DNA and histones are responsible for tissue-specific gene expression in eukaryotes and thus can lead to a change in an organism's phenotype (Gama-Sosa et al., 1983). If the environment induces epigenetic marks in a repeatable way and these marks are inherited across generations then there is potential for epigenetics to play important roles in organismal adaptation in natural populations (Gadjev, 2015), which can then affect the organism's interaction with other organisms. Therefore, we propose that epigenetic mechanisms may be important for the evolution of both insect-plant and insect-microbe interactions.

### Patterns of DNA methylation in different phyla

One type of epigenetic mark that is widespread and generally occurs across all domains of life is DNA methylation. DNA methylation involves the enzymatic addition of a methyl group to individual nucleotide bases of DNA in chromosomes by DNA methyltransferases (DMNTs). Now more than ever, DNA methylation has become more tractable to study due to the recent advancement in sequencing technology and bioinformatics. In turn the field of epigenomics is more accessible to researchers for both model and non-model organisms. As such the number of research articles on DNA methylation has been steadily increasing (Romanoski et al., 2015). For example, according to the Web of Science (2016), the number of epigenetic research articles has doubled every 5 years since 1971, reaching just over 110,000 articles between 2011 and 2015.

The role and patterns of DNA methylation vary widely among the three domains of life (Jeltsch, 2010). Several independent losses of DNA methylation have occurred in various eukaryotic taxa, suggesting that the role of DNA methylation may not be essential for all eukaryotic species (Field et al., 2004; Wion and Casadesús, 2006). For example, in the model systems *Caenorhabditis elegans* (roundworm) and *Drosophila melanogaster* (fruit fly) DNA methylation is not functional, because of lineage specific losses of DMNTs (Goll and Bestor, 2005). Nevertheless, DMNTs are present and DNA methylation is prevalent and functional in a wide diversity of other eukaryotic taxa including plants, vertebrates, and invertebrates (Jeltsch, 2010).

In vertebrates, including humans, cytosine methylation is widespread in the genome, specifically at cytosine-phosphate-guanine (CpG) dinucleotide sites (Bird, 1986). However, CpG-rich regions called CpG islands, which are typically 300- to 3000- base pairs long and are located primarily in the promoter regions of vertebrate genes, are largely un-methylated (Bird, 1986). Methylation of even a single CpG site in a promoter region can significantly inhibit transcription of the downstream genes (Robertson et al., 1995). Such transcriptional inhibition or gene silencing is an important role for DNA methylation as it also helps maintain the integrity of the genome by silencing transposable elements (Zamudio et al., 2015). DNA methylation also has a well-established role in imprinting, such as mammalian X-chromosome inactivation (Augui et al., 2011; Balaton et al., 2015), and differential expression of parental-specific alleles (Reik et al., 1987; Li et al., 1993; Razin and Cedar, 1994). Furthermore, DNA methylation in gene-body regions (e.g., un-translated regions, exons, and introns) can also affect the activity of genes in vertebrate genomes. For example, in human cell lines the inhibition of DNA methylation in gene-body regions resulted in the alternative splicing of exons (Maunakea et al., 2013).

In invertebrates, cytosine methylation also plays an important role in gene regulation. Epigenomic research in invertebrates initially lagged behind, because the two main invertebrate model species in genetics, *C. elegans* and *D. melanogaster*, do not have active copies of DNMTs (detailed above). Nevertheless, DNA methylation has been observed in a diversity of other invertebrate species. For example, in the Pacific oyster *Crassostrea gigas*, different levels of CpG methylation have been observed that correlate with gene functions (Gavery and Roberts, 2010). In the Chinese white shrimp, *Fenneropenaeus chinensis*, tissue-specific DNA methylation was observed (He et al., 2015). Furthermore, DNA methylation has been reported in many insect species of various orders including Diptera, Hemiptera, Hymenoptera, Lepidoptera, Coleoptera, Odonata, and Orthoptera (Field et al., 2004; Richards et al., 2008; Walsh et al., 2010; Xiang et al., 2010; Zhang J. et al., 2015; Zhang M. et al., 2015). In general, methylation levels of invertebrate CpG sites are relatively low, ranging from 0.36–20% (Regev et al., 1998), compared to mammalian systems where 60–90% of all CpG dinucleotides are subject to methylation (Suzuki and Bird, 2008).

Methylome studies across different invertebrate taxa have revealed that DNA methylation is often confined to genic regions (promoters, exons, and introns) of the genome, whereas intergenic regions remain largely unmethylated (Suzuki and Bird, 2008). In hymenopteran genomes, such as parasitoid wasps, ants, and bees, low levels of DNA methylation occur within transposable elements (TEs) compared to vertebrate genomes. These results suggest that DNA methylation has no or very little association with the repression of TEs as shown in vertebrates (Yan et al., 2015). DNA methylation within invertebrate genes has been associated with gene activation and alternative splicing. For example, loss of DNA methylation from multiple CpG sites within the insecticide-detoxifying esterase gene E4 of the green peach aphid *Myzus persicae* was associated with a reduction of transcription of the esterase gene E4, and thus increased sensitivity to pesticides (Field et al., 1989; Field, 2000). Also, several studies have proposed that DNA methylation is associated with alternative splicing of mRNA transcripts, which leads to behavioral regulation and caste specificity in eusocial insects including bees (Foret et al., 2012; Li-Byarlay et al., 2013), ants (Bonasio et al., 2012), and termites (Terrapon et al., 2014).

### Insect DNA methylation and adaptation to variable environments

Mounting evidence from the handful of non-model animal systems that have been studied suggests that environmental cues can trigger the reprogramming of cells through DNA methylation, resulting in the regulation of adaptive traits (Kucharski et al., 2008; Moczek and Snell-Rood, 2008; Alvarado et al., 2015; Table 1). As such, differential methylation patterns have the potential to produce an adaptive regulatory response to current environmental conditions. Environmental signals such as diet, stress, and anxiety have been shown to alter DNA methylation patterns during an organism's lifetime (Weaver et al., 2004; Jankard and Herman, 2008; Schwenk et al., 2013). For example, in the honey bee nutritional cues from royal jelly regulate queen determination via epigenetic mechanisms. Specifically, the gene, *dynactin p62* is differentially methylated in queens compared to workers, and it is hypothesized to be a key gene in regulating different developmental pathways (Kucharski et al., 2008). In this study, when DNA methyltransferase 3 (*Dnmt3*) is silenced in larvae that feed on protein-rich royal jelly these larvae develop into fertile queens with fully developed ovaries (Kucharski et al., 2008). This result indicates that a nutritional signal can alter epigenetic patterns resulting in caste determination. In another honeybee study, social stimuli of bees were highly correlated with changes in DNA methylation patterns between worker and nurse bees of the same age (Lockett et al., 2012). One particular CpG site in the gene Protein kinase C-binding protein 1 (*PKCbp1*) with variable levels of methylation between worker and nurse bees was strongly correlated with the alternative splicing of its gene product. The direct consequences of this alternative splicing however are unclear (Lockett et al., 2012). DNA methylation also plays a role in caste determination of another social hymenopteran, the carpenter ant *Camponotus floridanus*, by modifying ant body size, a key trait associated with the division of labor (Alvarado et al., 2015).

**Table 1:** **DNA methylation in various insects and its associated phenotypic effects**.

**Species**	**Common name**	**Phenotype**	**References**
*Acyrthosiphon pisum*	Pea aphid	Color morph differentiation	Dombrovsky et al., 2009
*Aedes aegypti*	Mosquito	Wolbachia infection and gene transcription	Ye et al., 2013
*Apis mellifera*	Honeybee	Caste determination	Elango et al., 2009; Foret et al., 2012; Herb et al., 2012; Patalano et al., 2012
*Apis mellifera*	Honeybee	Learning and memory processing	Lockett et al., 2010; Biergans et al., 2012
*Bombus terrestris*	Bumblebee	Reproductive caste formation	Kankanamge and Eranthi, 2015
*Bombyx mori*	Silkworm	Immune response against bacterial infection	Xiang et al., 2010; Zhang Q. L. et al., 2015
*Componotus floridanus*	Florida carpenter ant	Caste determination	Bonasio et al., 2012
*Coptotermes formosanus*	Subterranean termite	Gene regulation	Glastad et al., 2012
*Lucusta migratoria*	Migratory locust	Alternative migratory phenotypes	Robinson et al., 2015
*Medauroidea extradentata*	Stick insect	Gene regulation	Krauss et al., 2009
*Myzus persicae*	Peach-potato aphid	Upregulation of insecticide detoxifying esterases	Field et al., 1989; Hick et al., 1996; Field and Blackman, 2003
*Nasonia vitripennis*	Jewel wasp	Photoperiodic response on diapause	Werren et al., 2010; Park et al., 2011; Pegoraro et al., 2016
*Nasonia vitripennis*	Jewel wasp	Embryo development	Zwier et al., 2012
*Nilaparvata lugens*	Brown planthopper	Female fecundity	Zhang J. et al., 2015
*Onthophagus* sp.	Horned Beetle	Nutritional plasticity	Snell-Rood et al., 2012
*Pogonomyrmex barbatus*	Red harvester ant	Caste determination	Smith et al., 2012
*Reticulitermes flavipes*	Subterranean termite	Gene regulation	Glastad et al., 2012
*Schizaphis graminum*	Greenbug aphid	Upregulation of insecticide detoxifying esterases	Ono et al., 1999
*Sogatella furcifera*	Rice planthopper	Sexual dimorphism	Zhang M. et al., 2015
*Sogatella furcifera*	Rice planthopper	Wing dimorphism	Zhou et al., 2013
*Zootermopsis nevadensis*	Dampwood termite	Caste differentiation	Terrapon et al., 2014

In the parasitoid wasp *Nasonia vitripennis* changes in photoperiod are hypothesized to induce genome-wide DNA methylation changes (Pegoraro et al., 2016). When day length is decreased female *N. vitripennis* wasps induce developmental arrest of their progeny (diapause). This photoperiodic response allows the larvae to survive throughout winter. Knock-down of either DNMT1a or DNMT3 in *N. vitripennis* parents disrupted the photoperiod-induced developmental arrest of their larvae. Although the exact mechanisms are yet to be elucidated, these results suggest that environmentally induced diapause in *N. vitripennis* are linked to DNA methylation.

In addition to hymenopterans, methylation contributes to adaptive regulatory responses to environmental conditions in aphids. In the green peach aphid, *M. persicae*, individuals resistant to an organophosphate pesticide encode a differentially methylated esterase gene that confers the resistant phenotype (Field et al., 1989, 1996; Hick et al., 1996). Biotypes of the Russian wheat aphid, *Diuraphis noxia*, that differ in virulence toward their host plant display differential methylation patterns for key genes that are expressed in their salivary glands, suggesting that methylation may play an important role in this insect's ability to feed on different host plant cultivars (Gong et al., 2012). In *A. pisum*, it has been shown that extreme temperatures may result in variation in DNA methylation patterns, which are correlated to different color phenotypes within genetic clones (Dombrovsky et al., 2009). This study revealed that the intensity of methylation in CpG-islands within aphid cuticular genes varied dramatically between three different *A. pisum* color morphs (white, pink, and green). Furthermore, the authors identified correlations between CpG island methylation and growth rate, morph development, and pigmentation of the aphid population by pharmacologically inhibiting the DNA methyltransferases (Dombrovsky et al., 2009). In sum, DNA methylation may help drive rapid and precise gene regulation to variable environmental conditions.

### Role of DNA methylation in symbioses

In the sections above we detailed several examples of how environmental cues can induce specific DNA methylation patterns in insects, which can result in adaptive gene expression profiles. Although microbial associations are ubiquitous in many insect systems, as of yet, there has not been extensive research on how microbes affect insect host epigenomics. Nevertheless, we predict that epigenomics may play major roles broadly in insect-plant and insect-microbe ecology and evolution. For example, insect microbial associations have facilitated numerous host plant niche expansions, and the diversification of insect lineages (Hansen and Moran, 2014). Moreover, insect symbionts can contribute to a variety of extended insect host phenotypes, which include: defense against viral pathogens, fungal pathogens, and parasitoids (Kaltenpoth et al., 2005; Oliver et al., 2005; Scarborough et al., 2005; Scott et al., 2008; Teixeira et al., 2008; Vorburger et al., 2009), conferring thermal tolerance (Dunbar et al., 2007; Brumin et al., 2011), facilitating food digestion (Brownlie et al., 2009; Salem et al., 2012), and manipulating sexual reproduction (Stouthamer and Werren, 1993). Currently, we are still in the discovery phase in identifying specific genetic mechanisms that facilitate these important microbe-induced, insect-extended phenotypes.

To the best of our knowledge the only studies that demonstrate an effect of microbes on insect epigenomics are of *Wolbachia* and mosquitos. *Wolbachia* is an intracellular bacterial symbiont that is both vertically and horizontally inherited in numerous insect species and commonly enhances its transmission through reproductive manipulations (Stouthamer and Werren, 1993). Nevertheless, mosquitos like *Drosophila*, do not have functional DNA methylation, because they do not encode DMNT1 and DMNT3 (Holt et al., 2002; Nene et al., 2007). However, they do encode the methyltransferase, DMNT2, which has substrate specificity for tRNAs (Goll et al., 2006), contributes to antiviral defense in *Drosophila* (Durdevic et al., 2013), and is involved in random genome methylation patterns (Kunert et al., 2003). In one study, when the pathogenic strain of *Wolbachia* (*w*MelPop) infects the mosquito, *Aedes aegypti*, the mosquito is hypomethylated because *Wolbachia* suppresses DNMT2 (Zhang G. et al., 2013). When DMNT2 is overexpressed in mosquito cell lines *Wolbachia* replication is inhibited, suggesting that suppression of DNMT2 is beneficial for *Wolbachia* survival. Conversely, in *A. aegypti* DNMT2 is induced by the Dengue virus, and this induction promotes Dengue virus replication. *In vivo* this antagonistic interaction ultimately results in *Wolbachia* suppressing the Dengue virus via DNMT2 suppression (Zhang G. et al., 2013). In another study, the *Wolbachia* strain *w*MelPop results in both the methylation and de-methylation of *A. aegypti's* genome (Ye et al., 2013). For the most part these changes in methylation primarily appeared to be random (Ye et al., 2013). In this study, the direct effect of differential methylation on transcription in *w*MelPop-infected compared to uninfected mosquitos remains unclear.

Given the paucity of evidence of symbionts affecting invertebrate host epigenetics and studies of insect hosts with functional DNA methylation systems investigating the effect of symbionts on vertebrate hosts may provide insights into possible ways symbionts may impact DNA methylation in insect genomes. In general, DNA methylation in vertebrate studies of gut-associated microbes has revealed that host immune responses and microbially derived metabolites affect host DNA methylation. For example, in mice Takahashi et al. (2011) found that the methylation level of the Toll-like receptor 4 gene in intestinal epithelial cells is significantly lower in germ-free mice compared to conventional mice. Moreover, results from this study suggests that this epigenetic modification is elicited by and important for the maintenance of commensal microbes in the gut. In another study, when the human pathogen *Helicobacer pylori* infects the human gut DNA methylation increases in the promoter regions of the human genes filamin C and thrombomodulin. This results in the silencing of these genes and a concomitant increase in the risk of gastric cancer (Nakajima et al., 2009). In another human microbiome study, an increase in abundance of two members of the human oral microbiome that belong to Enterobacteriaceae and Tenericutes is associated with the hypermethylation of the promoter regions of the human host gene *MDR1*. Hypermethylation of *MDR1* can result in head and neck squamous cell carcinoma (Bebek et al., 2012). In another study on host pathogens, pathogenic viruses including human adenovirus, hepatitis B virus and HIV are known to increase genome-wide levels of methylation of their host by up-regulating DNMT1 (Fang et al., 2001; Burgers et al., 2007; Jung et al., 2007).

In addition to pathogenic and non-pathogenic gut microbes mediating human immune responses through DNA methylation, microbe-derived metabolites can also influence DNA methylation in humans and ultimately impact expressed phenotypes. For example, nutritional uptake in early postnatal humans modifies the infant's gut microbiome, which in turn affects the epigenetic patterns of the individual (Mischke and Plosch, 2013). This study proposes that changes in the composition of the gut microbiome results in altered profiles of microbe-produced metabolites such as folate and short-chain fatty acids. The same study proposed that an increase in such metabolites may influence the DNA methylation patterns of adjacent intestinal cells, which in turn results in the predisposition to obesity.

Microbial symbionts and pathogens of humans and some insects have been demonstrated to alter patterns of DNA methylation. As such microbial symbionts have the capacity to (radically) alter host phenotypes. We hypothesize that this ability is widespread in insect symbionts, particularly among co-evolved insect symbionts. These intimate partners may influence methylation of their insect hosts with functional DNA methylation systems by modulating their host's immune responses to microbes. For example, attenuating immune responses so as to permit their intracellular persistence. In addition, these symbionts can encode novel biosynthetic pathways, which may contribute microbially derived metabolites, such as folate; folate is a key source of the one carbon group used to methylate DNA (Table 2). Moreover, in co-evolved insect symbioses tissue- specific, DNA-methylation patterns in specialized insect cells that harbor obligate symbionts may facilitate the development and regulation of this long-term symbiotic relationship. Nevertheless, our understanding of the development and regulation of these symbiotic cells in insects is still nascent (Braendle et al., 2003; Hosokawa et al., 2016). Therefore, by investigating if and how epigenetic modifications affect the regulation of insect-microbe interactions, we will gain a better understanding of key biological mechanisms in symbiosis and evolution in general.

**Table 2:** **Presence and absence of pathways from obligate symbionts of insects with fully sequenced genomes**.

**Insect host**	**Microbe symbiont**	**Folate biosynthesis**	**Methionine metabolism**	**Sulfur metabolism**	**Genome size (Mbp)**
*Coptotermes formosanus*	*Azobacteroides pseudotrichonymphae*	Complete	Partial	Partial	1.22
*Graphocephala atropunctata*	*Baumannia cicadellinicola BGSS*	Complete	Absent	Complete	0.64
*Camponotus chromaiodes*	*Blochmannia chromaiodes*	Complete	Complete	Complete	0.79
*Camponotus floridanus*	*Blochmannia floridanus*	Complete	Complete	Complete	0.71
*Camponotus pennsylvanicus*	*Blochmannia pennsylvanicus*	Complete	Complete	Complete	0.79
*Camponotus vafer*	*Blochmannia vafer*	Complete	Complete	Complete	0.72
*Heteropsylla texana*	*Carsonella ruddii HT*	Absent	Absent	Absent	0.16
*Heteropsylla cubana*	*Carsonella ruddii HC*	Absent	Absent	Absent	0.17
*Ctenarytaina spatulata*	*Carsonella ruddii CS*	Absent	Absent	Absent	0.16
*Ctenarytaina eucalypti*	*Carsonella ruddii CE*	Absent	Absent	Absent	0.16
*Pachypsylla venusta*	*Carsonella ruddii PV*	Absent	Absent	Absent	0.16
*Pachypsylla celtidis*	*Carsonella ruddii PC*	Absent	Absent	Absent	0.16
*Bemisia tabaci*	*Portiera aleyrodidarum*	Absent	Absent	Absent	0.35
*Acyrthosiphon pisum*	*Buchnera aphidicola APS*	Partial	Absent	Complete	0.66
*Acyrthosiphon kondoi*	*Buchnera aphidicola AK*	Partial	Absent	Complete	0.65
*Uroleucon ambrosiae*	*Buchnera aphidicola UA*	Partial	Absent	Complete	0.63
*Schizaphis graminum*	*Buchnera aphidicola Sg*	Partial	Absent	Complete	0.64
*Baizongia pistaciae*	*Buchnera aphidicola Bp*	Partial	Absent	Absent	0.62
*Cinara tujafilina*	*Buchnera aphidicola Ct*	Absent	Absent	Absent	0.44
*Aspidiotus nerii*	*Uzinura diaspidicola*	Partial	Absent	Absent	0.26
*Planococcus citri*	*Tremblaya princeps*	Absent	Absent	Absent	0.14
*Planococcus citri*	*Moranella endobia*	Absent	Absent	Absent	0.54
*Homalodisca vitripennis*	*Sulcia muelleri Hc*	Absent	Absent	Absent	0.15
*Diceroprocta semicincta*	*Sulcia muelleri DSEM*	Absent	Absent	Absent	0.28
*Clastoptera arizonana*	*Sulcia muelleri CARI*	Absent	Absent	Absent	0.28
*Megacopta punctatissima*	*Ishikawaella capsulata*	Complete	Complete	Partial	0.75
*Diceroprocta semicincta*	*Hodgkinia cicadicola*	Absent	Partial	Absent	1.11
*Graphocephala atropunctata*	*Baumannia cicadellinicola BGSS*	Complete	Absent	Partial	0.76
*Macrosteles quadrilineatus*	*Nasuia deltocephalinicola*	Absent	Partial	Absent	0.11
*Pediculus humanus*	*Riesia pediculicola*	Complete	Absent	Absent	0.58
*Blattella germanica*	*Blattabacterium* sp. *Bge*	Complete	Partial	Partial	0.64
*Mastotermes darwiniensis*	*Blattabacterium* sp. *MADAR*	Complete	Absent	Partial	0.59
*Cryptocercus punctulatus*	*Blattabacterium* sp. *Cpu*	Complete	Absent	Absent	0.61
*Blaberus giganteus*	*Blattabacterium* sp. *BGIGA*	Complete	Partial	Absent	0.63
*Panesthia angustipennis spadica*	*Blattabacterium* sp. *BPAA*	Complete	Partial	Absent	0.63
*Diaphorina citri*	*Profftella armatura*	Absent	Absent	Partial	0.46
*Glossina morsitans morsitans*	*Wigglesworthia glossinidia*	Complete	Absent	Absent	0.72

“*Complete” denotes that all of the enzymes for a particular pathway are encoded in the genome. “Partial” denotes that only some enzymes for a particular pathway are encoded in the genome. “Absent” denotes that no orthologs of the enzymes of a particular pathway are present in the genome. Information for gene presence and absence is based on GenBank, KEGG, and BioCyc data*.

*The selected pathways produce important microbially derived metabolites that may influence insect host DNA methylation patterns*.

## Gene regulation via small RNAs

In recent years, there has been a rapid increase in the identification of non-coding, regulatory RNAs in Bacteria, Archaea, and Eukaryotes (Babski et al., 2014). With the improvement of sequencing technology it has been found that the non-coding RNAs expressed within in a cell include more than just the rRNAs, tRNAs, small nuclear RNAs (snRNAs), and small nucleolar RNAs (snoRNAs). What has become clear is that these non-coding RNAs of varying sizes are important in a myriad of biological functions, which includes gene regulation. One large class of non-coding RNAs is regulatory small RNAs (sRNAs). sRNAs can be categorized depending on their function, structure, conservation among taxa, and/or size range (Kim et al., 2009; Waters and Storz, 2009). In general, the term sRNAs encompasses a large diversity of expressed RNAs that have various size cut offs depending on what domain you are studying (Babski et al., 2014). This review will broadly define sRNAs as regulatory elements that are transcribed from coding and/or non-coding regions, which have both perfect and/or imperfect base-pairing interactions with their target RNA, and are <300 nt in length.

### Bacterial sRNAs

In general, sRNAs fall into two categories: *cis*-encoded and *trans*-encoded sRNAs. Within bacteria, *trans*-encoded sRNAs are encoded at genomic locations that are distant from their target mRNA(s), as such they often have partial complementarity with their targets. *Trans*-encoded sRNAs are generally dependent on the RNA chaperone Hfq (Storz et al., 2011). Hfq proteins protect sRNAs from ribonuclease degradation and facilitate intermolecular contacts between sRNAs and their mRNA targets in the Enterobacteriaceae (reviewed in Vogel and Luisi, 2011; Sauer, 2014). Despite its important role, Hfq cannot be detected in some bacterial phyla including the Chlamydiae, Spirochaetae, Actinobacteria, Deinococcus-Thermus, Cyanobacteria, Chlorobi, and Bacteroidetes (Sun, 2002). In the Firmicutes, a gram-positive bacterial phylum, some species encode Hfq, however, unlike the Enterobacteriaceae, it does not play a major role in gene regulation (reviewed in Bouloc and Repoila, 2016). There is evidence that some of these gram-positive bacteria have analogous proteins that carry out similar roles to Hfq (reviewed in Durand et al., 2015). In many insect endosymbionts belonging to gram-negative bacteria, the homolog of Hfq has been lost (Sun, 2002). This suggests *trans*-encoded sRNAs are not expressed, and/or if they are expressed, other stabilizing mechanisms are employed (Hansen and Degnan, 2014).

Currently, *trans*-encoded sRNAs are the most extensively characterized sRNAs in bacteria. This is because of the historical difficulties that arose when trying to identify *cis*-encoded sRNAs (Georg and Hess, 2011). Improvements in sequencing technologies and methodologies, and bioinformatics prediction programs have led to an increase in the identification of candidate *cis*-encoded sRNAs, however validating the functional role of many of these accumulating candidates still lags behind (Barquist and Vogel, 2015). *Cis*-encoded sRNAs can be transcribed in the 5′ and/or 3′ regions of a coding sequence, within the coding sequence, or within the non-coding regions, and have perfect complementarity with their target RNAs. Many *cis*-encoded sRNAs in free-living bacteria have been observed to have a variety of roles, such as expression within mobile genetic elements like plasmids, transposons and phage, aiding in mRNA stability and degradation, and/or translational inhibition and attenuation (Wagner and Simons, 1994; Brantl, 2007). In addition, *cis*-encoded sRNAs have been identified to inhibit the synthesis of toxic proteins (Fozo et al., 2008).

Riboswitches are another common bacterial sRNA. Riboswitches are characterized by having complex folded domains—“the riboswitch,” and they encompass a non-coding portion of the mRNA that binds to various metabolites (Nahvi et al., 2002; Winkler et al., 2002; Lai, 2003; Winkler and Breaker, 2003). In the presence of specific metabolites, riboswitches undergo a conformational change which then influences transcription, translation, or other processes related to protein production (Mandal and Breaker, 2004). Early surveys of riboswitches found that within many gram-positive bacteria such as the Firmicutes, riboswitches tended to modulate transcriptional attenuation, whereas in gram-negative bacteria such as the Proteobacteria, many of the riboswitches identified regulated translational attenuation (Nudler and Mironov, 2004; Barrick and Breaker, 2007). However, as riboswitches are described in more species, the regulation of the riboswitch seems to be more closely associated to ligand/type of riboswitch (Ray and Chakdar, 2015).

### Eukaryotic sRNAs

In general, eukaryotic sRNAs can be divided into two families: micro RNAs (miRNAs) and small-interfering RNAs (siRNAs). Within eukaryotes, the RNAi pathway is responsible for the regulation of a diversity of endogenous genes via miRNAs, and for protecting the organism from invasive genetic material, such as viruses and transposable elements via siRNAs (Hannon, 2002). These sRNAs act as template RNA that bind to the RNA-induced silencing complex (RISC), and degrade complementary RNA sequences (Hannon, 2002). This pathway is conserved across most eukaryotes, however it has been lost in some fungal lineages and, notably, in the model system, *Saccharomyces cervisiae* (Billmyre et al., 2013).

miRNAs are ~21–24 nucleotides in length and are transcribed from genes or within introns and subsequently form hairpin structures that are cleaved to result in the final mature miRNA (Ha and Kim, 2014). In animals, miRNAs share partial sequence complementarity to multiple target mRNAs, much like bacterial *trans*-encoded sRNAs. Plant miRNAs on the other hand have high sequence complementarity (Jones-Rhoades et al., 2006). Among plants and animals, miRNAs mediate post-transcriptional gene regulation in various shared aspects of physiology and development (Flynt and Lai, 2008; de Lima et al., 2012; Vidigal and Ventura, 2015).

Unlike miRNAs, siRNAs are ~21 nt long and have endogenous or exogenous origins such as transposons and viruses. Furthermore, they are formed from double- stranded RNAs (Carthew and Sontheimer, 2009; Piatek and Werner, 2014). Also unlike miRNAs, siRNAs generally share perfect complementarity with their targets. Animal siRNAs are important in regulating transposons, heterochromatic sequences, intergenic regions, long RNA transcripts with extensive structure, and mRNAs (Ghildiyal and Zamore, 2009). It has been found that many expressed endo-siRNAs have vital roles in animal development (reviewed in Ghildiyal and Zamore, 2009). Within plants, there are three types of endo-siRNAs: *cis*-acting siRNAs (casiRNAs), *trans*-acting siRNAs (tasiRNAs), and natural antisense transcript-derived siRNAs (natsiRNAs; reviewed in Ghildiyal and Zamore, 2009; Axtell, 2013; Pattanayak et al., 2013). In general these endo-siRNAs are important in methylation (Mette et al., 2000; Llave et al., 2002; Tran et al., 2005), development (Allen and Howell, 2010), modulation of the plant's responses to stress (Jones-Rhoades and Bartel, 2004; Sunkar and Zhu, 2004; Borsani et al., 2005; Fujii et al., 2005) and plant immunity (Katiyar-Agarwal et al., 2006).

In both plants and animals, miRNAs and siRNAs share biosynthesis pathways that include RNA endonucleases Argonaute proteins and proteins Drosha and Dicer (Carthew and Sontheimer, 2009; Piatek and Werner, 2014). Though the genes involved in the biosynthesis of these sRNAs may share a common ancestor (Shabalina and Koonin, 2008), plant and animal sRNAs differ in biogenesis, function, and subsequent evolutionary trajectories (Shabalina and Koonin, 2008; Axtell et al., 2011; Mukherjee et al., 2013).

### Evolution of small RNAs in reduced genomes

Intracellular symbionts, pathogens, and organelles are subject to repeated genetic bottlenecks, which contribute to a suite of associated genome-wide changes including genome-size reduction. This evolutionary constraint also results in biases in nucleotide composition (increased %A+T), elevated mutation rates and the fixation of deleterious and neutral mutations due to genetic drift, ultimately resulting in gene inactivation and loss (Moran et al., 2009; Moran and Bennett, 2014). Moreover, selection becomes less effective in maintaining the mechanisms of gene regulation in reduced genomes compared to free-living relatives with larger genomes and access to more diverse environments (Lambert and Moran, 1998; Hansen and Moran, 2012; Hansen and Degnan, 2014). Thus, over evolutionary time, genome sizes tend to shrink dramatically, losing numerous functional capabilities and becoming structurally rearranged compared to free-living relatives (Moran and Bennett, 2014).

Despite these dramatic changes, reduced genomes still display strong purifying selection for key genes that are essential for the host-microbe relationship, including core housekeeping genes (Moran et al., 2009; Williams and Wernegreen, 2011). Among these are genes encoding biologically active non-coding RNAs, such as tRNAs and rRNAs that display higher %G+C composition relative to coding sequences, and accumulate compensatory base substitutions to maintain important secondary structural regions (e.g., stems; Lambert and Moran, 1998; Hansen and Moran, 2012). The increased genome wide %A+T composition in reduced genomes results in numerous polyA and polyT homopolymers. Such homopolymers are readily subject to insertions or deletions (indels) that can disrupt promoters or result in non-functional proteins because of frame shifts (Dunbar et al., 2007). These indels can initiate the processes of genome erosion (Moran et al., 2009) but in several instances transcriptional slippage can occur at these polyA/T tracts, restoring the reading frame of the mRNA and yielding functional proteins (Tamas et al., 2008; Wernegreen et al., 2010). Importantly, transcriptional slippage has been: (i) observed in symbionts with reduced genomes, (ii) has been maintained over evolutionary time, and (iii) functionally has been shown to rescue the activity of important genes for the symbiont's lifestyle (Tamas et al., 2008; Wernegreen et al., 2010). Collectively these patterns indicate that even though these reduced genomes experience severe genetic drift, purifying selection is strong enough to maintain and/or co-opt compensatory mechanisms to maintain fundamental processes for the persistence of microbe lineages.

Thus, we hypothesize that bacterial symbiont genomes subject to radical gene loss and structural rearrangements over evolutionary time can compensate for the loss of *essential* regulatory machinery by maintaining and/or co-opting compensatory mechanisms, such as sRNAs to aid in the regulation of vital symbiotic genes and core housekeeping processes, as suggested in Hansen and Degnan (2014).

sRNAs in Bacteria and Eukaryotes have been associated with rapid diversification and adaptation of lineages to local environments (Horler and Vanderpool, 2009; Yu et al., 2010; Ames and Lovell, 2011; He et al., 2011; Raghavan et al., 2012). It has been suggested that sRNAs can rapidly evolve from non-adaptive transcripts (Raghavan et al., 2012). Non-adaptive transcripts, i.e., transcriptional noise, can result when RNA polymerase accidentally recognizes non-promoter sequences as promoter sequences, because promoters contain low information content (Mendoza-Vargas et al., 2009; Raghavan et al., 2012). In reduced genomes that experience severe genetic drift, it is unclear how novel regulatory sRNAs could evolve. However, given that purifying selection has been identified in these reduced genomes for both coding and non-coding functional RNA sequences (Lambert and Moran, 1998; Moran et al., 2009; Williams and Wernegreen, 2011; Hansen and Moran, 2012), we hypothesize that purifying selection is strong enough to maintain and/ or co-opt emergent regulatory sRNAs that are essential for the symbiont's gene regulation. The failure to maintain such essential sRNAs would be lethal for the microbe and in turn its host, if other host and/or microbe factors failed to compensate.

One example of how reduced genomes compensate to maintain essential gene expression processes comes from previous research on *Buchnera* from several diverse aphid species (Hansen and Moran, 2012). In this study, the authors revealed that *Buchnera* tRNA genes in four divergent taxa are often shorter in length than their homologs in *E. coli, Buchnera*'s distant free-living relative. The difference in length is typically three nucleotides, and mostly reflects the loss of the encoded 3′ CCA sequence in the *Buchnera* tRNA genes. At the 3′ end of tRNAs, the nucleotide sequence CCA is required for amino acid activation and must either be encoded in the tRNA gene or added during tRNA maturation by the CCA-adding enzyme (*cca*). In *Buchnera*'s close relatives, such as *E. coli, Vibrio* spp., and *Pseudomonas* spp., 3′ CCA is encoded in all tRNA genes that are present in *Buchnera*; most likely to maintain efficient translation. Nevertheless, only half of *Buchnera* tRNA genes encode 3′ CCA (Hansen and Moran, 2012). Importantly, using directional RNAseq, Hansen and Moran (2012) found that mature RNA transcripts of these genes possess a CCA at the 3′ end, implying CCA-addition and thus activation of amino acids. The role of the CCA-adding enzyme in *E. coli* and other organisms that already encode CCA in their tRNA gene sequences is to monitor the stability of tRNAs by tagging unstable tRNAs with an additional CCA for degradation (Wilusz et al., 2011; Kuhn et al., 2015). In turn, this study revealed that *Buchnera* is compensating for the loss of CCA in the tRNA gene sequence by co-opting the CCA-adding enzyme. If *Buchnera* did not co-opt this regulatory machinery for this additional role, which is not observed in *Buchnera*'s closest free-living relatives, then the symbiont would not be able to translate proteins. Moreover, the authors observed numerous compensatory base substitutions in tRNA stems and high %GC in tRNA genes compared to coding sequences that help maintain tRNA secondary structure and function. In sum, these results provide evidence that purifying selection is strong enough in reduced genomes to (1) co-opt existing machinery to compensate for core regulatory processes and (2) maintain existing machinery to conserve essential regulatory functions.

### sRNAs in organelle genomes

Organelles represent the most dramatic example of tiny genomes that express regulatory sRNAs. Within eukaryotes there are two general types of organelles that have a unique evolutionary history with a microbial origin, such as mitochondria and plastids (Smith and Keeling, 2015). Mitochondria and plastids arose from bacteria that were once endosymbionts in early host eukaryotic cells (reviewed in Katz, 2012). In lineages that harbor both types of organelles, acquisition of plastids occurred after the early mitochondrial-eukaryotic lineage diversification (Gould et al., 2008). Mitochondria are believed to have evolved from a single endosymbiotic event involving an Alphaproteobacterium most likely from a Rickettsiales ancestor (Williams et al., 2007). Plastids on the other hand have a more complicated evolutionary history. All plastids evolved from the primary endosymbiosis of a cyanobacterium; however, across lineages there have also been secondary and tertiary endosymbiotic events (Gould et al., 2008). Currently, there is a growing body of research focused on organelle-associated sRNAs and their regulation (Table 3). Overall there is an emerging trend that gene regulation within the organelle is controlled by both host nuclear-encoded sRNAs and organelle-encoded sRNAs.

**Table 3:** **Summary of studies isolating mitochondrial/plastid-encoded sRNAs and nuclear-encoded sRNAs found within organelles**.

**Study**	**Organism**	**Organelle**	**sRNAs identified**
**ORGANELLE-ENCODED sRNAs**			
Lung et al., 2006	*Nicotiana tabacum*; Leaves	Plastid	*Ntc-1, Ntc-2, Ntc-3, Ntc-4, Ntc-5, Ntc-6, Ntc-7, Ntc-8, Ntc-9, Ntc-10, Ntc-11, and Ntc-12*
	Mouse liver and kidney	Mitochondria	*Mt-1, Mt-2, Mt-3, Mt-4, Mt-5, and Mt-6*
Mercer et al., 2011	Human 143B cells	Mitochondria	31 (26 mapping to tRNAs)
Smalheiser et al., 2011	Mouse hippocampus	Mitochondria	18 (9 mapping to a tRNA)
Sripada et al., 2012	HEK293 and HeLa	Mitochondria	miR-4461, miR-4463, miR-4484, miR-4485, and 7 punitive miRNA
Ro et al., 2013	Human	Mitochondria	2540 miRNAs
	Mouse	Mitochondria	1499 miRNAs
Zhou et al., 2014	*Laodephax striatellus*	Mitochondria	3977 [mRNAs (1546), tRNAs (308), and rRNAs (2091)]
Wu et al., 2015	*Silene noctiflora*	Mitochondria	9 miRNAS
**NUCLEAR-ENCODED sRNAs ISOLATED WITH MITOCHONDRIA**			
Kren et al., 2009	Rat liver	Mitochondria	*mirR-130a/b, mirR-140, mirR-320, mirR-494, mirR-671, mirR-202, mirR-763, mirR-198, mirR-765, mirR-705, mirR-709, mirR-721, and mirR-761*
Bian et al., 2010	mouse liver	Mitochondria	Top 20 expressed: *miR-122, miR-805, miR-690, miR-494, miR-705, miR-721, miR-720, miR-188-5p, miR-101, miR-let-7f, miR-711, miR-432, miR-181b, miR-361-5, miR-680, miR-181d, miR-29c, miR-29a, and miR-762*
Barrey et al., 2011	Human skeletal muscle myoblasts	Mitochondria	*miR-720, miR-133b, miR-1974, miR- 24, miR-133a, miR-125a-5p, miR-1979, miR-103, miR-125b, miR-103, miR-221, miR-23a, miR-let-7b, miR-423-3p, miR- 106a, miR-23b, miR-92a, miR-193b, and miR-365*
Bandiera et al., 2011	HeLa cells	Mitochondria	*miR-1973, miR-1275, miR-494, miR-513-a-5p, miR-1246, miR-328-5p, miR-1908, miR-1972, miR-1977, miR-638, miR-1974, miR-1978, and miR-1201*
Sripada et al., 2012	HEK293 and HeLa	Mitochondria	209 nuclear coded with punitive and 230 references in miRBase
**KNOWN FUNCTIONS OF NUCLEAR SRNAS ON MITOCHONDRIAL BIOLOGY AND METABOLISM**			
**Study**		**Organelle process**	**sRNA**
Aoi et al., 2010; Li et al., 2010; Wang J.-X., et al., 2011; Wang et al., 2012; Yamamoto et al., 2012; Kang et al., 2013; Long et al., 2013; Zhang Y. et al., 2013; Li J. et al., 2014; Li X. et al., 2014; Tak et al., 2014		Mitochondrial fission and biogenesis	*miR-696, miR-30, miR-499, miR-484*, miR-494, *miR-761, miR-106b, miR-140, miR-196, and miR-27a/b*
Zhu et al., 2009; Zhang et al., 2010; Frankel et al., 2011; Xiao et al., 2011		Mitophagy	*miR-101, miR-204, miR-30a, and miR-21*
Aschrafi et al., 2008; Gao et al., 2009; Fang et al., 2012; Jiang et al., 2012; Latronico and Condorelli, 2012; Sun et al., 2012; Bienertova-Vasku et al., 2013; Das et al., 2014; Tomasetti et al., 2014		Mitochondrial metabolism	*miR-155, miR-143, miR-326, miR-124, miR-137, miR-340, miR- 183, miR-743a, miR-181c, miR-210, miR-338, miR-14, miR-15b, miR-16, miR-195, miR-424 miR-338, and miR-23a/b*

*Nuclear-encoded sRNAs have been found to affect various aspects of organelle biology. Included are examples of the sRNAs that regulate genes affecting mitochondrial biogenesis and fusion, mitophagy, and metabolic functions*.

In the early 2000's it was thought that few if any sRNAs were expressed within organelle genomes (e.g., Lung et al., 2006). However, the identification of sRNAs has increased rapidly over the last decade for uncultivable organelles and microbes, because of lower sequencing costs and advances in sequencing technology (Hotto et al., 2011; Wang L. et al., 2011; Ro et al., 2013; Wu et al., 2015). There are two types of sRNAs identified within organelles: nuclear-encoded sRNAs, that are eukaryotic-like in origin and organelle-encoded sRNAs, that are bacterial-like in origin. Currently, the method by which nuclear-encoded sRNAs are transported into the organelle is unclear. However, there is evidence to suggest that tRNAs and 5S rRNAs can be imported into mammalian mitochondria and indirect evidence that tRNAs also can be imported into plastids (Schneider, 2011). In turn, similar pathways may be used for sRNA uptake into organelles. It is important to note that not only are nuclear-encoded sRNAs present within organelles, but argonaute (specifically, Ago2), the nuclear-encoded protein of the RISC complex, is also present within mitochondria (Bian et al., 2010; Bandiera et al., 2011). The presence of Ago2 and both nuclear-encoded and organelle-encoded sRNA within organelles supports the hypothesis that there is a complex interplay of gene regulation between the host and the organelle.

In mammals, besides providing energy for cellular functioning, mitochondria are also important in apoptosis (Lee et al., 2004; Suen et al., 2008), calcium concentration regulation (O'Rourke, 2004; Chen et al., 2005), and reactive oxygen species production (Chen et al., 2003). As such, in mammals, mitochondrial dysfunction has been linked to various diseases (Au et al., 2005; Baloyannis, 2006; Lemieux et al., 2010; Ritov et al., 2010; Gong et al., 2011). Some mitochondrial disease phenotypes are associated with the dysregulation of mitochodrial sRNAs (mitomiRs; Table 3). In general, there is less work reported in plant organelle sRNAs (Budak and Akpinar, 2015). Wang L. et al. (2011) is one of the few studies that show that chloroplast-encoded sRNAs can respond to environmental changes in the Chinese cabbage (*Brassica rapa* ssp. *Chinensis*). From these studies, we can see a potential trend emerging that organelle sRNAs expressed either from the organelle genome or the nuclear genome of their hosts are important for the regulation of these small, eroded genomes.

### sRNAs in reduced bacterial genomes

Overall, organelles, especially animal mitochondria, share many genomic characteristics to endosymbionts: organelles have reduced genomes when compared to free living relatives (Green, 2011; Gray, 2012), are generally low in GC content (McCutcheon and Moran, 2012; Smith, 2012), and their operons are highly fragmented (Barbrook et al., 2010; Brinza et al., 2010; Hansen and Degnan, 2014). Unlike organelles, most intracellular symbionts and pathogens with reduced genomes have not transferred regulatory genes to their host's chromosomes and still have autonomous housekeeping functions (Bennett and Moran, 2013).

Within bacterial genomes, operon structure, regulatory proteins, and sigma factors govern gene regulation. Organisms that possess reduced genomes, such as intracellular pathogens and mutualistic symbionts have lost many of these hallmark regulatory features (e.g., *Mycoplasma* sp. and *Buchnera*; Dandekar et al., 2000; Shigenobu et al., 2000). Within the human pathogen, *Mycoplasma pneumoniae*, it has been revealed that ~13% of coding genes have a corresponding antisense RNA (Güell et al., 2009). The function of a few of these antisense sRNA candidates has been determined and have been shown to down-regulate their target gene (Güell et al., 2009). Two of these sRNAs, NEW87 and NEW8, potentially have roles in metabolism and DNA repair and replication, respectively (Güell et al., 2009). Several of these antisense sRNAs are conserved in the closely related species *Mycoplasma genitalium*, however, the functionality of these sRNA transcripts including NEW87 and NEW8 have not yet been validated (Lluch-Senar et al., 2007). There is evidence that suggests that many of the transcripts that are found within reduced bacterial genomes are simply the by-product of transcriptional noise (Raghavan et al., 2012; Llorens-Rico et al., 2016), highlighting the importance of validating the function of identified sRNAs.

Another type of intracellular parasite, *Rickettsia*, is an intracellular alphaproteobacterial endosymbiont that is found widely within arthropod lineages. The most widely studied *Rickettsia* species cause vertebrate diseases such as Typhus and Rocky Mountain spotted fever (Weinert et al., 2009). These pathogenic species also spend at least part of their lifecycles in arthropod vectors. A recent study observed sRNA expression within 13 *Rickettsia* species and identified 1785 novel sRNAs (Schroeder et al., 2015). The number of sRNAs identified within each species varied from 15–191 and there was no correlation with genome size and number of sRNAs identified (Schroeder et al., 2015).

*Wolbachia*, another intracellular alphaproteobacterium, which is closely related to *Rickettsia*, is found within many insect taxa and other invertebrates (Hilgenboecker et al., 2008). It has varied effects on its host, such as reproductive manipulation (Werren et al., 2008), inhibition of vector-bone pathogen transmission (Moreira et al., 2009; Bian et al., 2013), and as a symbiont providing vitamins to its host (Hosokawa et al., 2010). When *Wolbachia* parasitically infects mosquito (*A. aegypti*) cell lines, it has been found to express sRNAs that potentially increase bacterial fitness by upregulating host genes that help facilitate its own replication (Mayoral et al., 2014a). It has been found that *Wolbachia* infection of *A. aegypti* results in differential expression of host miRNAs compared to uninfected control mosquitos (Hussain et al., 2011; Mayoral et al., 2014b). Hussain et al. (2011) identified that the host sRNA, aae-miR-2940, which upregulates a metalloprotease, is necessary for successful *Wolbachia* infection.

Until recently, there was little evidence that gene regulation occurred within unculturable mutualistic endosymbionts. It has been hypothesized that symbionts like *Buchnera*, display minimal gene regulation because they live in a stable intracellular environment (Shigenobu et al., 2000). *Buchnera* microarray experiments have provided limited evidence that this endosymbiont is able to regulate the transcription of genes underlying essential amino-acid (EEA) pathways in response to aphid nutritional demand (Moran et al., 2003, 2005; Reymond et al., 2006; Viñuelas et al., 2011). In turn, *Buchnera*'s gene regulation at the RNA level is generally assumed to be non-existent (Hansen and Moran, 2014). However, a recent study showed that *Buchnera* exhibits differential expression of 80 proteins between two different *Buchnera* life-stages, including proteins that are involved in essential amino acid biosynthesis with no concomitant changes in mRNA expression suggesting that post-transcriptional regulation was occurring (Hansen and Degnan, 2014). To support this hypothesis, Hansen and Degnan (2014), also found that across highly divergent *Buchnera* lineages, there is widespread conservation of *cis*- and/or *trans*-encoded sRNAs. They also revealed that of the 80 differentially expressed proteins identified between the two *Buchnera* life stages, 86% have evidence of possible *cis*-acting regulatory sRNAs (Hansen and Degnan, 2014). Many of the differentially expressed proteins in EEA pathways also had *cis* sRNAs identified within their transcriptional unit or flanking them (Hansen and Degnan, 2014). These observations led the authors to hypothesize that these patterns in differential protein expression may result from post-transcriptional regulation, such as conserved sRNAs (Hansen and Degnan, 2014). Based on this study's results, we predict that these novel sRNAs potentially have important regulatory roles, especially for *Buchnera's* EAA pathways.

In summary, there is growing evidence that both organelles and bacteria with highly reduced genomes, have maintained key sRNAs to aid in the regulation of genes that are vital to their core biological functioning. Organelles have not only evolved mechanisms to uptake vital eukaryotic nuclear encoded sRNAs but also the necessary machinery (Ago2) to process these sRNAs. There is evidence that shows that bacteria can produce sRNAs that target eukaryotic genes, suggesting that these sRNAs either mimic eukaryotic sRNAs or can be co-opted into the RNAi pathway. This type of trans-kingdom communication via sRNAs has also been observed in fungal plant pathogens (Weiberg et al., 2013) and the malarial parasite (LaMonte et al., 2012). There is also evidence demonstrating that organelles have maintained sRNAs within their highly eroded bacterial genome. Within endosymbionts, there is evidence that sRNAs are important for regulating functions important in maintaining the symbiosis with their host. Overall, these examples of sRNA utilization supports the hypothesis that within organisms that have undergone radical gene loss, sRNAs may aid in the regulation of vital symbiotic genes and core housekeeping processes.

## Future directions and conclusions

Recent studies on epigenomic and small-RNA regulation in eukaryotes and bacteria have challenged historical preconceptions of how these genomes are regulated and evolve. The implications of these new studies on the understanding of the regulation of shared animal-microbe metabolic processes warrant further investigation in future studies. Such studies are critical because of the importance these regulatory mechanisms have in the evolution and maintenance of these widespread herbivore symbioses. To this end, we conclude this review by proposing future directions that will aid in teasing apart the regulatory underpinnings and dynamics of shared metabolic processes that are ubiquitous between insect herbivores and their microbial symbionts.

### Epigenomics in insect-plant interactions

Elucidating the mechanisms and patterns of DNA methylation and its inheritance is important for understanding the molecular biology, ecology, and the evolution of non-model animals. For example, methylation patterns are linked not only to numerous human diseases (Robertson, 2001; Richardson, 2003; Lund et al., 2004; Stenvinkel et al., 2007; Mastroeni et al., 2010; Benton et al., 2015) but also to insect and mammalian behavior (Kucharski et al., 2008; Dias and Ressler, 2014; Alvarado et al., 2015), and pesticide resistance in insects (Field et al., 1989, 1996; Hick et al., 1996; Table 1). Some of these epigenetic-associated phenotypes are inherited from parent to offspring (Dias and Ressler, 2014), which influences current views of the mechanisms of evolution, particularly the central dogma (Szyf, 2014). CpG DNA methylation is present in divergent eukaryotic taxa such as fungi, animals, and plants (Wang et al., 2006; Feng et al., 2010; Zemach et al., 2010), and therefore its effects on gene regulation and organismal ecology and evolution are of broad interest to science in general.

Aphids are an attractive and tractable model for studying epigenetic regulation of shared herbivore-microbe metabolisms. Aphids display functional DNA methylation (Walsh et al., 2010) in contrast to many classical model organisms (e.g., *D. melanogaster, S. cervisiae, C. elegans*; Goll and Bestor, 2005; Feng et al., 2010). Moreover, *A. pisum* can be reared in culture, maintained asexually or sexually, have short generation times, have co-evolved microbial symbionts, and exhibit a wide range of host plant interactions. Thus, aphids are ideally suited for characterizing novel aspects of epigenetic regulation. Results produced from this research can thus be important for understanding insect nutrition specifically, as well as the ecology and evolution of insect-host plant interactions and symbiosis more generally. In addition, many obligate symbionts in insect systems produce folate and/or other methyl- groups, which may influence insect host DNA-methylation patterns (Table 2). In turn, more work is needed on the epigenetics of animal-microbe interactions, especially for non-classical model insect systems. Currently, many of the outstanding questions on this topic (Table 4) can now be addressed using cutting-edge molecular genetics, genomics, and bioinformatics tools.

**Table 4:** **Outstanding questions in regard to the relative importance of regulatory mechanisms that are key to shared herbivore-microbe metabolisms**.

**EPIGENETIC REGULATORY MECHANISMS**
Can co-evolved insect symbionts modulate their host's immune responses toward them by influencing their host's methylation patterns in symbiotic host cells?
Can insect symbionts modulate their host's DNA-methylation patterns by producing folate and/or methy-groups for their host?
Is DNA inside of specialized insect host cells that harbor symbionts (e.g., bacteriocytes) differentially methylated compared to DNA in other body tissues? If so, is this linked to gene expression patterns inside of these specialized cells?
When insect herbivores feed on different host plants do different DNA methylation profiles result in specialized insect symbiont cells? If so, are these inherited and thus can they dynamically influence host plant adaptation?
Are methylation patterns conserved in specialized insect symbiont cells for insect orthologs that are hypothesized to play a conserved core role in the shared insect-microbe metabolism?
Can environmental stimuli, such as host plant nutritional quality, trigger a regulated metabolic response via DNA methylation to compensate for deficient nutrients?
**sRNA REGULATORY MECHANISMS**
Are post-transcriptional gene expression processes widespread in obligate symbionts of sap-feeding insects?
In addition to *Buchnera*, are conserved sRNAs expressed in other obligate symbiont lineages of sap-feeding insects?
What are the functions of conserved sRNAs in obligate symbionts of sap-feeding insects? Are they important in the regulation of essential amino acid pathways and if so can they respond to aphid nutrient demand?
How do sRNAs evolve in reduced bacterial genomes?

### Small RNAs in insect-plant interactions

Insect taxa in several of the most diverse insect orders are known to harbor obligate symbionts with reduced genomes. Most of these symbionts have been characterized in blood and sap-feeding insects; the smallest known bacterial genome is a symbiont from a sap-feeding insect (Bennett and Moran, 2013). Similar to organelles, such as mitochondria, these obligate symbiont genomes are greatly reduced due to genetic drift, primarily encode genes essential for their host, are unculturable, and have lost most genes for transcriptional regulation. Nevertheless, unlike organelles these symbionts encode core housekeeping genes, and in general none of its genes were transferred to the host's chromosome (Bennett and Moran, 2013). To this end obligate symbionts like the model *Buchnera* are still autonomous bacterial cells, however some of these minimal genomes can regulate gene expression at the post-transcriptional level (Hansen and Degnan, 2014). Potentially in these small genomes the loss of canonical regulatory proteins has resulted in the evolution of compensatory regulatory mechanisms, such as small regulatory RNAs (Hansen and Degnan, 2014). In sum, if these genomes rely primarily on small RNAs for gene regulation, instead of proteins, this could be a prime example of how genomes revert to the “RNA world” for gene regulation. More functional and comparative genomic studies using novel manipulative techniques on unculturable microbes are required to further understand the putative regulatory role of sRNAs in symbionts with reduced genomes. Ultimately, these future studies will determine the relative importance of these microbial regulatory mechanisms in these intimate symbioses. Outstanding questions on this topic are presented in Table 4 and can now be addressed using classical microbiology techniques, cutting-edge genomics, and bioinformatics tools.

## Author contributions

DK, MT, and AH all substantially wrote and edited the manuscript.

### Conflict of interest statement

The authors declare that the research was conducted in the absence of any commercial or financial relationships that could be construed as a potential conflict of interest.
